# Effects of late gestational nutrient restriction on uterine artery blood flow, placental size, and cotyledonary mRNA expression in primiparous beef females

**DOI:** 10.1093/jas/skae145

**Published:** 2024-05-24

**Authors:** Colby A Redifer, Lindsey G Wichman, Abigail R Rathert-Williams, Allison M Meyer

**Affiliations:** Division of Animal Sciences, University of Missouri, Columbia, MO 65211, USA; Division of Animal Sciences, University of Missouri, Columbia, MO 65211, USA; Division of Animal Sciences, University of Missouri, Columbia, MO 65211, USA; Division of Animal Sciences, University of Missouri, Columbia, MO 65211, USA

**Keywords:** beef heifers, developmental programming, Doppler ultrasonography, pregnancy, undernutrition, uteroplacenta

## Abstract

Fall-calving primiparous beef females [body weight (BW): 451 ± 28 (SD) kg; body condition score (BCS): 5.4 ± 0.7] were individually-fed either 100% (control; CON; *n* = 13) or 70% (nutrient restricted; NR; *n* = 13) of metabolizable energy and metabolizable protein requirements for maintenance, pregnancy, and growth from day 160 of gestation to parturition. Doppler ultrasonography of both uterine arteries was conducted pre-treatment and every 21 d from days 181 to 265 of gestation. Expelled placentas were collected, and ipsilateral cotyledonary tissue was sampled to assess relative messenger ribonucleic acid (mRNA) expression. Placentas were separated into ipsilateral and contralateral sides, dissected (cotyledonary vs. intercotyledonary), and dried. Data were analyzed with nutritional plane, treatment initiation date, and calf sex (when *P *< 0.25) as fixed effects. Uterine blood flow included day and nutritional plane × day as repeated measures. We previously reported that post-calving, NR dams weighed 64 kg less and were 2.0 BCS lower than CON, but calf birth weight was not affected. Maternal heart rate was less (*P* < 0.001) for NR dams than CON after nutritional planes began. Nutritional plane did not affect (*P *≥ 0.20) uterine artery hemodynamics, but all variables were affected (*P *≤ 0.04) by day. Contralateral cotyledonary and placental weight were less (*P* ≤ 0.04) and contralateral intercotyledonary weight and number of cotyledons tended to be less (*P* ≤ 0.10) for NR dams than CON, but ipsilateral and whole placental weights were not affected (*P *≥ 0.13). Ipsilateral placental weight as a percentage of total placental weight was greater (*P* = 0.03) for NR dams than CON. Whole placental cotyledonary: intercotyledonary weight was less (*P* = 0.01) for NR dams than CON. Placental efficiency was not affected (*P* = 0.89) by nutritional plane. Cotyledonary relative mRNA expression of GLUT3 and SNAT2 was greater (*P* ≤ 0.05) and relative expression of GLUT1, GLUT4, and NOS3 tended to be greater (*P* ≤ 0.07) for NR dams than CON. Nutritional plane did not affect (*P *≥ 0.13) relative mRNA expression of GLUT5, 4F2hc, CAT1, LAT1, LAT2, VEGFA, FLT1, KDR, GUCY1B3, and PAG2. Despite less contralateral placental growth, beef heifers experiencing late gestational nutrient restriction maintained uterine artery blood flow and total placental mass and had 4 nutrient transporters and 1 angiogenic factor upregulated in cotyledons, all of which likely contributed to conserving fetal growth.

## Teaser

First-parity beef females that were nutrient restricted during late gestation maintained similar total uterine artery blood flow, dry total placental weight, and calf birth weight compared with controls. Nutrient restriction decreased contralateral placental growth, but placental adaptation included greater expression of 4 nutrient transporters and 1 angiogenic factor in ipsilateral cotyledons.

## Introduction

During late gestation, maternal energy and protein requirements increase substantially in the beef female to support exponential growth of the uteroplacenta, fetus, and mammary gland (Ferrell et al., 1976; NASEM, 2016). The placenta serves as the interface for the exchange of nutrients, gases, and wastes between maternal and fetal circulations, functions as a highly active endocrine organ, and modulates the maternal immune response to prevent conceptus rejection (Carter, 2012). Monosaccharides and amino acids are transported across the placenta to the fetus for tissue accretion or to serve as oxidative fuel, but the placenta also requires an appreciable amount of nutrients itself (Ferrell et al., 1983). Thus, adequate fetal growth is dependent on successful placental nutrient transport capacity which involves factors such as available nutrients in maternal circulation, blood flow to the gravid uteroplacenta, placental size, abundance of nutrient transporters, and placental morphology (Fowden et al., 2006). Rarely are many of these interrelated factors characterized in the same experiment, and pregnancies are frequently terminated for sample collection before the completion of gestation (Lemley et al., 2018; Contreras-Correa et al., 2021; Swanson et al., 2022); thus, it is unusual for these factors to be studied when calves are also followed postnatally.

Poor maternal nutrition during pregnancy can result in placental insufficiency and intrauterine growth restriction of the offspring, increase the likelihood of neonatal mortality, and developmentally-program postnatal productivity (Wu et al., 2006; Reynolds et al., 2010b). The first-parity beef female is still growing during gestation and establishing a successful gravid uterus for the first time; thus, there is competition for nutrients between fetal, uteroplacental, mammary, and maternal tissue growth (Short and Adams, 1988; Holland and Odde, 1992). Primiparous beef females have decreased placental mass and smaller calf birth weight but increased placental mass relative to maternal body weight than multiparous beef females, demonstrating that the first pregnancy is challenged irrespective of maternal nutrition (Meyer and Redifer, 2024). There is a critical need to evaluate the effects of late gestational undernutrition in first-parity beef females on the many factors involved in placental nutrient transport capacity collectively, in a model where viable, full-term calves can be followed for postnatal objectives.

Data reported here are from an intensive experiment (Redifer et al., 2023) to determine the effects of late gestational nutrient restriction in individually-fed first-parity beef females on prenatal and postnatal nutrient availability and utilization by their calves. We hypothesized that late gestational nutrient restriction decreases blood flow to the uterus, impairs placental growth, and reduces cotyledonary nutrient transporter and angiogenic factor abundance, ultimately impairing placental nutrient transport capacity to the developing fetus. Our objectives were to investigate the effects of late gestational nutrient restriction in primiparous beef females on uterine artery blood flow, expelled placental size, and cotyledonary relative mRNA expression of nutrient transporters and angiogenic factors.

## Materials and Methods

All procedures were approved by the University of Missouri Animal Care and Use Committee (Protocol #9877), and research was conducted at the University of Missouri Beef Research and Teaching Farm (Columbia, MO).

### Animal management and diets during gestation

Animal management and nutritional plane treatments applied during late gestation have been described previously (Redifer et al., 2023). Heifers were bred to a single Angus sire via artificial insemination, then transrectal ultrasonography was utilized to confirm pregnancy approximately 35 d after artificial insemination with fetal sex determined between days 60 and 80 of gestation.

Twenty-six single-sired fall-calving Hereford × Simmental-Angus crossbred beef heifers [initial body weight (**BW**) = 451 ± 28 (SD throughout methods) kg, initial body condition score (**BCS)** = 5.4 ± 0.7, calving at 2 yr of age] were allocated by BW, BCS, fetal sex, and expected calving date to 1 of 2 late gestational nutritional planes from day 160 of gestation to parturition. Control (**CON**; *n* = 13) heifers were individually-fed 100% of estimated metabolizable energy (**ME**) and metabolizable protein (**MP**) requirements for maintenance, pregnancy, and growth, whereas nutrient restricted (**NR**; *n* = 13) heifers were individually-fed 70% of ME and MP requirements. Heifers were housed in 12 partially-covered 3.7 × 15.8 m pens (*n* = 2 or 3 per pen), penned by nutritional plane, and individually-fed via a Calan gate feeding system (Calan Broadbent Feeding System, American Calan, Northwood, NH).

Nutrient requirements were estimated using an expected calf birth weight of 34 kg and projected maternal average daily gain of 0.36 kg/d. Metabolizable energy for maintenance was based on data for heifers in confinement (0.138 Mcal ME/kg non-gravid BW^0.75^; Freetly and Hales, personal communication). The equation used for ME for conceptus was published previously (Freetly et al., 2005). Equations from NASEM (2016) were utilized for ME for gain and MP for maintenance, conceptus, and gain. Nutrient requirements were adjusted weekly using the most recent dam BW (recorded every 21 d) and day of gestation.

From days 160 to 265 of gestation, diets were based on ad libitum chopped sorghum sudan hay [1.74 Mcal ME/kg, 6.69% crude protein (**CP**), 72.0% neutral detergent fiber (**NDF**), 52.8% acid detergent fiber (**ADF**); dry matter (**DM**) basis]. Starting on day 266 of gestation, a 3-d transition to ad libitum chopped endophyte-infected tall fescue-based hay (1.90 Mcal ME/kg, 7.22% CP, 65.1% NDF, 43.2% ADF; DM basis) occurred to allow for less supplement needed to meet estimated nutrient requirements for the end of gestation and upcoming lactation. Using expected individual hay intakes (estimated from the previous week’s hay intakes), heifers were supplemented daily with whole corn, dried distillers’ grains with solubles (**DDGS**), and soyhull pellets to meet their assigned nutritional plane. Supplement for each heifer was formulated and weighed individually. Dams had ad libitum access to water and a trace mineralized salt block (Big 6 Mineral Salt, Compass Minerals America Inc., Overland Park, KS).

### Uterine artery blood flow

Uterine blood flow was determined before nutritional plane treatment allocation (day 157 of gestation) and at 21-d intervals occurring on days 181, 202, 223, 244, and 265 of gestation (collected within ± 2 d of the actual day of gestation). Hemodynamics of the ipsilateral and contralateral uterine arteries were assessed via transrectal color Doppler ultrasonography using an Aloka Prosound α6 (Hitachi Aloka, Tokyo, Tokyo, Japan) equipped with a 7.5 MHz convex finger transducer (Aloka USD-995) using methods similar to those described by Camacho et al. (2014).

With the ultrasound in B-mode with color Doppler, the probe was inserted through the rectum in a dorsal view to locate the bifurcation of the internal and external iliac arteries from the aorta. The internal iliac artery was traced caudally, and the uterine artery [sometimes referred to as the common trunk of the uterine and umbilical arteries (Getty, 1964; Dyce and Wensing, 1971)] was identified as the first major descending artery to branch off the internal iliac artery. On each side, the uterine artery was confirmed as a movable artery within the mesometrium and with increasing fremitus (fluid turbulence associated with a buzzing sensation) as pregnancy advanced. The uterine artery was measured immediately distal to branching off the internal iliac artery to ensure that measurements were made at the same location along the vessel for all animals and always measured proximal to the umbilical artery branching off the true uterine artery. The umbilical artery is obliterated in the adult, has a very small lumen, and has minimal blood flow which supplies the bladder (May, 1970; Dyce and Wensing, 1971); thus, it has negligible contribution to uterine blood flow measurements.

**Figure 1.**
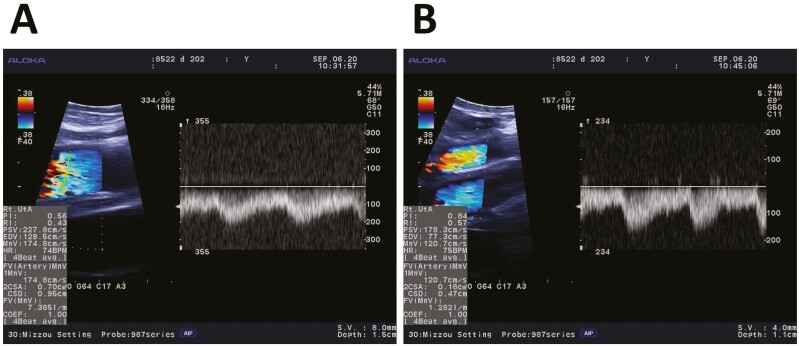
Color Doppler sonograms of the ipsilateral (Panel A) and contralateral (Panel B) uterine arteries of a primiparous beef female on day 202 of gestation. The images were obtained chute side in B|D mode using an Aloka Prosound α6 machine. In B mode (left side), Doppler flow gain was set at 40, the angle of insonation (68° or 69°, respectively) was set parallel to the walls of the artery in a longitudinal view, and the caliper function was used to measure the cross-sectional diameter of the artery perpendicular to the angle of insonation. In D mode (right side), 4 representative and consistent cardiac waveforms were selected (only 3 are shown), from a similar set of frames as those from which the vessel was chosen. The machine calculated all variables, as displayed at the bottom left.

The finger transducer was oriented so that a longitudinal section of the uterine artery could be visualized and maintained at the largest cross-sectional diameter. At that time, the ultrasound machine was switched to a split screen view so that the longitudinal section of the artery and cardiac waves could be viewed simultaneously (Figure 1). After a consistent set of cardiac waves and a clear view of the vessel were obtained, the screen was paused for data collection.

On each ultrasound day, 3 or 4 ultrasound scans (each scan including ≥3 cardiac cycle waveforms) were obtained for the ipsilateral and contralateral uterine arteries, with all measurements made and data recorded chute-side. If after 3 scans were conducted, angle of insonation, mean velocity, cross-sectional area, or blood flow from 1 scan was inconsistent with the other 2, then a 4^th^ scan was conducted, and either all 4 scans were kept, or the inconsistent scan was removed. For all uterine blood flow data collection, a single trained technician conducted the scans, and a separate single trained technician captured the measurements on the ultrasound machine. All ultrasound examinations were conducted between 0800 and 1400 h and lasted approximately 40 min for each female. Once an animal was in the chute, aerosol insecticide was applied to prevent animal discomfort, and a 24-in. livestock fan was used to provide heat abatement when necessary.

The average angle of insonation for obtaining blood flow waveforms was maintained between 58° and 80° (average: 71.8°) for the ipsilateral side and between 57° and 79° (average: 65.7°) for the contralateral side. The average angle of insonation from previous scans was used to maintain consistency of angle within each side for a single female across all 6 timepoints. The average angles of insonation for the ipsilateral and contralateral uterine arteries were not affected (*P* ≥ 0.22) by the late gestational nutritional plane × day of gestation interaction or the main effect of nutritional plane, but both were affected (*P* ≤ 0.01) by day of gestation. Flow gain was set between 40 and 45 (average: 40.8) for the ipsilateral side and between 40 and 55 (average: 42.7) for the contralateral side.

Maternal heart rate, peak systolic velocity, end diastolic velocity, mean velocity, pulsatility index, resistance index, cross-sectional diameter, cross-sectional area, and blood flow were recorded for each side separately. The Doppler software was preprogrammed to calculate pulsatility index = (peak systolic velocity − end diastolic velocity)/ mean velocity; resistance index = (peak systolic velocity − end diastolic velocity)/ peak systolic velocity; and blood flow (L/min) = mean velocity (cm/s) × cross-sectional area (cm^2^) × 60 (s/min)/1,000. All uterine blood flow variables are reported for the ipsilateral and contralateral uterine arteries separately, except maternal heart rate which was averaged for the 2 sides. Total uterine blood flow for each female was calculated as the sum of the blood flow of the ipsilateral and contralateral uterine arteries.

### Expelled placental collection and processing

Beginning on day 274 of gestation, heifers were closely monitored 24 h/d by trained personnel to ensure that time of calving was observed. Dams were continuously monitored post-calving until placentas were collected after natural expulsion. Within 10 min of collection, 2 representative ipsilateral cotyledons that were centrally located and of average size and normal color were identified. Tissue from both cotyledons was excised, cleaned with phosphate-buffered saline as necessary, pooled, flash frozen on dry ice (2 to 5 g), and stored at −80°C until ribonucleic acid (**RNA**) extraction. Time from parturition to cotyledonary tissue sampling was not different (*P* = 0.39; 4.87 vs. 4.10 ± 0.61 h; range: 2.3 to 12.1 h) between late gestational nutritional planes.

**Figure 2.**
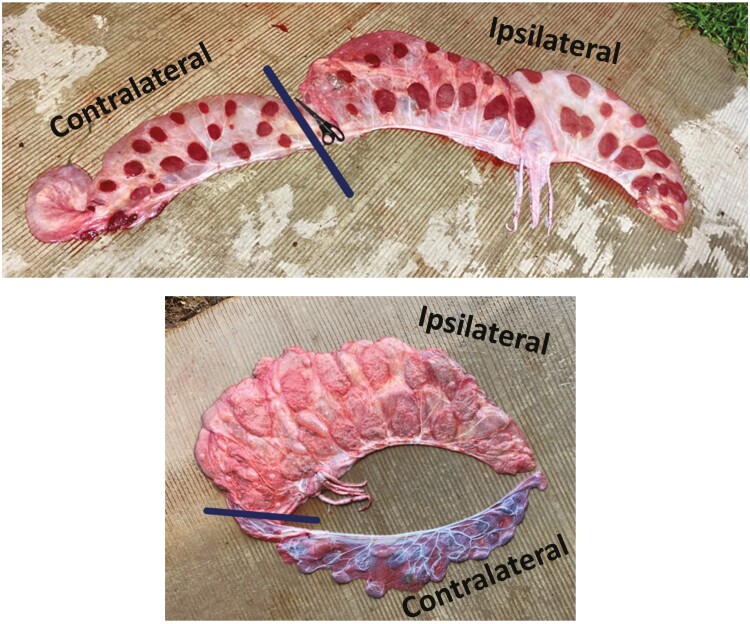
Representative images of expelled whole bovine placentas prior to being separated into ipsilateral and contralateral sides. The solid line indicates where placentas were separated based on anatomical landmarks and observations.

Placentas were rinsed thoroughly, determined to be complete, and separated into ipsilateral and contralateral sides to be dissected. The calf expulsion site from the placenta was an ideal landmark for dividing sides. The ipsilateral and contralateral sides were of similar length, the umbilical vessels were roughly centered on the length of the ipsilateral side, and occasionally the color of the cotyledons or the direction cotyledons were facing (inward or outward) differed between the sides for further confirmation (Figure 2).

Placenta sides were refrigerated, dissected, and stored at −20°C until all dams had calved, then DM weights were determined as described previously (Redifer et al., 2021). Dry matter was determined separately for cotyledonary and intercotyledonary tissues of the ipsilateral and contralateral sides, resulting in 4 parts per whole placenta. The 2 cotyledons from which tissue was sampled for RNA extraction were included in ipsilateral cotyledon number and cotyledonary DM (remaining tissue).

### Cotyledonary relative mRNA expression

A Cellcrusher (Cellcrusher, Portland, OR) was chilled on dry ice and used to pulverize cotyledonary tissue. Representative cotyledonary tissue (20 to 30 mg) was homogenized in buffer RLT Plus (lysis buffer) using a rotor-stator homogenizer with QIAshredder (Qiagen, Hilden, Germany), then total RNA was extracted using the RNeasy Mini Kit (Qiagen) following manufacturer instructions. Ribonucleic acid quantity (average: 277 ng/µL, range: 154 to 432 ng/µL) was assessed using a NanoDrop 1000 Spectrophotometer (Thermo Fisher Scientific Inc., Middleton, VA), and quality (average: 6.9 RNA integrity number, range: 5.7 to 8.7 RNA integrity number) was measured on an Agilent TapeStation 2200 (Agilent Technologies Inc., Santa Clara, CA). Subsamples of extracted RNA were pooled to use as an internal control, and all samples were stored at −80°C until complimentary deoxyribonucleic acid (**cDNA**) was synthesized from 1,000 ng of total RNA using the iScript cDNA Synthesis Kit (Bio-Rad Laboratories, Hercules, CA) following manufacturer instructions.

Optimal cDNA dilution and amplification efficiencies were determined for each primer using a 4-fold dilution series of cDNA, and each primer amplified at 98 to 106% efficiency. Optimal cDNA dilution was determined to be 1:5 in DNase/RNase-free water, and samples were stored at −20°C until quantitative real-time polymerase chain reaction (**qRT-PCR**). Bovine primer sequences for genes involved in placental glucose and fructose transport [GLUT1 (SLC2A1), GLUT3 (SLC2A3), GLUT4 (SLC2A4), and GLUT5 (SLC2A5)], amino acid transport [4F2hc (SLC3A2), CAT1 (SLC7A1), LAT1 (SLC7A5), LAT2 (SLC7A8), and SNAT2 (SLC38A2)], angiogenesis VEGFA (vascular endothelial growth factor A), NOS3 (nitric oxide synthase 3), FLT1 (VEGFA receptor 1), KDR (VEGFA receptor 2), and GUCY1B3 (nitric oxide receptor)], PAG2 (pregnancy-associated glycoprotein 2), and G3PDH (glyceraldehyde 3-phosphate dehydrogenase) were obtained from Bio-Rad Laboratories (PrimePCR SYBR Green Assay).

Analysis of messenger RNA (**mRNA**) expression was completed by qRT-PCR using SYBR Green Supermix (Bio-Rad Laboratories) and the QuantStudio 3 Real-time PCR System (Applied Biosystems, Grand Island, NY), with 20 µL total reaction volume for all genes. Each 96-well PCR plate contained a pooled internal control and a no template control (DNase/RNase-free water substituted for cDNA) to ensure there was no contamination, with all samples analyzed in duplicate. The qRT-PCR conditions were set as follows: 1 cycle at 95°C for 30 s, 40 cycles at 95°C for 15 s followed by 60°C for 30 s, followed by a melting curve program (from 65 to 95°C). Target mRNA cycle threshold values for each gene were normalized to a reference gene (G3PDH) with mRNA expression calculated relative to the pooled internal control using the 2^−ΔΔCT^ method (Livak and Schmittgen, 2001). Cycle threshold values for G3PDH were not affected (*P* = 0.32; 23.5 vs. 23.8 ± 0.2) by late gestational nutritional plane. For mRNA expression data, if a 2^−ΔΔCT^ was > 3 SD away from the group mean, it was considered an outlier (1 CON for NOS3, 1 CON for PAG2) and removed from the dataset. The intraassay and interassay CV for the pooled internal controls were 0.3 and 1.3%, respectively.

### Statistical analysis

One dam (CON) was completely removed from the study due to late gestational abortion. Uterine blood flow was not measured for 1 female (CON) because abnormal vasculature made it difficult to consistently detect blood flow in the contralateral uterine artery. For 1 female (CON), uterine blood flow was not determined on day 265 of gestation due to fetal crowding. Placentas retained for > 1 wk (1 CON and 1 NR) were not dissected for placental size upon natural expulsion, but for the CON female, ipsilateral cotyledons were excised from the placenta 12 h post-calving for qRT-PCR. One female (NR) retained her placenta for approximately 20 h post-calving, and it was dissected for placental size, but cotyledons were not sampled for qRT-PCR. One placenta (NR) was damaged at collection and the ipsilateral and contralateral sides were not easily discerned; thus, only whole placental weights were determined.

Uterine artery blood flow, placental size, and cotyledonary relative mRNA expression were analyzed using the MIXED procedure in SAS 9.4 (SAS Institute Inc., Cary, NC) with late gestational nutritional plane as a fixed effect and animal as the experimental unit. Uterine blood flow analyses also included day of gestation and the nutritional plane × day interaction as fixed effects. These were considered repeated measures using the majority best-fit covariance structure (based on Akaike Information Criterion, Bayesian Information Criterion, and corrected Bayesian Information Criterion) specific for each variable (chosen from unstructured, compound symmetry, heterogeneous compound symmetry, autoregressive, and heterogeneous autoregressive).

For all measures, Julian date of treatment initiation (to remove variation in breeding date and age of dam) and calf sex (if *P* < 0.25) were included as covariates (fixed effects). For cotyledonary relative mRNA expression, time from parturition to sampling was also included as a covariate. Significance was considered when *P* ≤ 0.05 and tendencies were considered when 0.05 < *P* ≤ 0.10. In the absence of interactions, main effects were reported. Means were separated using least significant difference and the same *P*-value thresholds.

## Results

### Uterine artery blood flow

**Figure 3.**
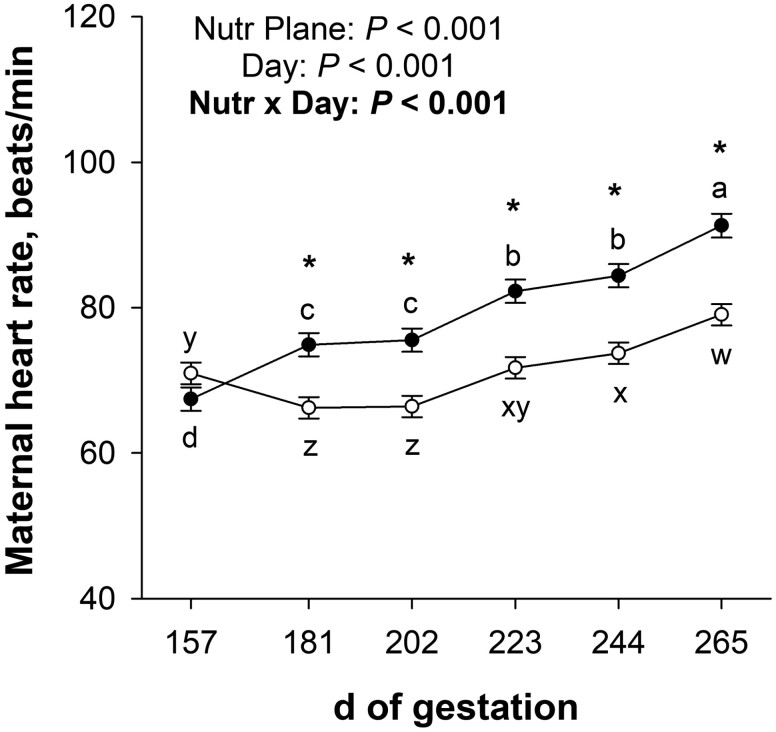
Effects of late gestational nutritional plane on maternal heart rate from days 157 to 265 of gestation. Solid circles represent primiparous beef females individually-fed 100% (Control; *n* = 10 to 11) and open circles represent primiparous beef females individually-fed 70% (Nutrient Restricted; *n* = 13) of metabolizable energy and metabolizable protein requirements for maintenance, pregnancy, and growth from day 160 of gestation to parturition. Least squares means ± SEM are presented. * Nutritional plane means within day differ (*P* ≤ 0.05). ^abcd^ Means differ (*P* ≤ 0.05) for Control across days. ^wxyz^ Means differ (*P* ≤ 0.05) for Nutrient Restricted across days.

There was a nutritional plane × day interaction (*P* < 0.001; Figure 3) for maternal heart rate during late gestation. Maternal heart rate was not affected (*P* = 0.11) by nutritional plane on day 157 of gestation (before nutritional plane treatment initiation) but was less (*P* < 0.001) for NR dams than CON from days 181 to 265 of gestation. For CON dams, maternal heart rate increased (*P* < 0.001) from days 157 to 181, from days 202 to 223, and from days 244 to 265 of gestation. For NR dams, maternal heart rate decreased (*P* < 0.001) from days 157 to 181 of gestation but increased (*P* < 0.001) from days 202 to 223 and from days 244 to 265 of gestation.

**Figure 4.**
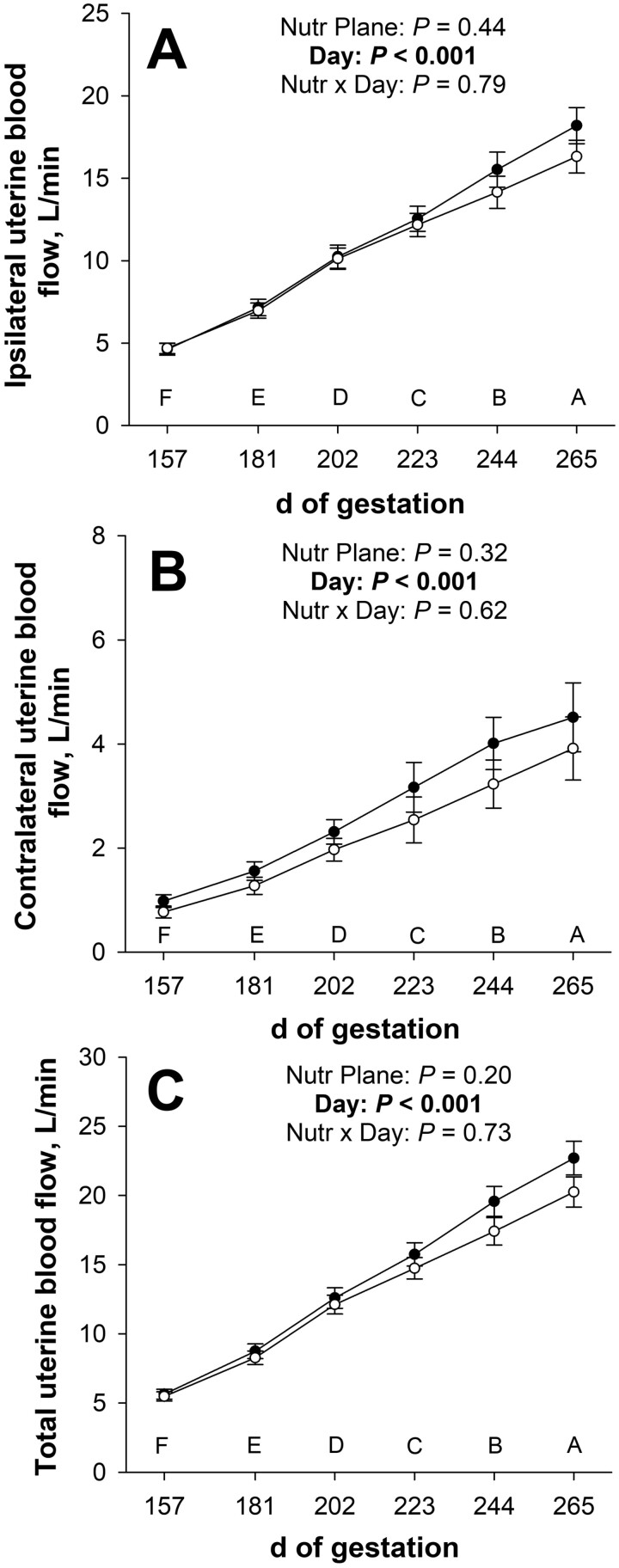
Effects of late gestational nutritional plane on ipsilateral (Panel A), contralateral (Panel B), and total (Panel C) uterine artery blood flow from days 157 to 265 of gestation. Solid circles represent primiparous beef females individually-fed 100% (Control; *n* = 10 to 11) and open circles represent primiparous beef females individually-fed 70% (Nutrient Restricted; *n* = 13) of metabolizable energy and metabolizable protein requirements for maintenance, pregnancy, and growth from day 160 of gestation to parturition. Least squares means ± SEM are presented. ^ABCDEF^ Means for main effect of day differ (*P* ≤ 0.05).

The late gestational nutritional plane × day of gestation interaction and the main effect of nutritional plane did not affect (*P* ≥ 0.20; Table 1) any uterine artery hemodynamics, but all variables were affected (*P* ≤ 0.04) by day of gestation. Peak systolic velocity, end diastolic velocity, and mean velocity of the ipsilateral uterine artery increased (*P* ≤ 0.04) from days 157 to 202 but did not change (*P* ≥ 0.23) from days 202 to 265 of gestation. Pulsatility index of the ipsilateral uterine artery decreased (*P* = 0.01), and resistance index tended to decrease (*P* = 0.06) from days 157 to 181 of gestation but did not change (*P* ≥ 0.24) from days 181 to 265 of gestation. Ipsilateral uterine artery cross-sectional area and blood flow increased (*P* < 0.001; Table 1; Figure 4) from days 157 to 265 of gestation. Ipsilateral uterine artery blood flow as a percentage of total blood flow was less (*P* ≤ 0.04) on days 223, 244, and 265 of gestation than on days 157 and 181 of gestation.

**Table 1.** Effects of late gestational nutritional plane and day of gestation of primiparous beef females on uterine artery blood flow

	Nutritional plane^1^			Day of gestation							*P*-value^2^		
Item	CON	NR	SEM^3^	157	181	202	223	244	265	SEM^3^	Nutr	Day	Nutr × Day
*Ipsilateral uterine artery*													
Peak systolic velocity, cm/s	322	312	15	274^c^	295^b^	336^a^	324^a^	335^a^	341^a^	13	0.63	< 0.001	0.68
End diastolic velocity, cm/s	163	161	7	137^c^	152^b^	175^a^	169^a^	170^a^	169^a^	6	0.78	< 0.001	0.65
Mean velocity, cm/s	234	226	10	191^c^	212^b^	244^a^	239^a^	245^a^	250^a^	9	0.51	< 0.001	0.52
Pulsatility index	0.674	0.672	0.018	0.709^a^	0.658^b^	0.658^b^	0.653^b^	0.673^ab^	0.688^ab^	0.018	0.96	0.04	0.77
Resistance index	0.487	0.484	0.009	0.493^ab^	0.474^b^	0.474^b^	0.479^b^	0.490^ab^	0.502^a^	0.009	0.82	0.03	0.82
Cross-sectional area, cm^2^	0.808	0.810	0.023	0.430^f^	0.580^e^	0.721^d^	0.893^c^	1.038^b^	1.192^a^	0.037	0.97	< 0.001	0.53
Blood flow, L/min	11.38	10.74	0.61	4.66^f^	7.06^e^	10.19^d^	12.36^c^	14.84^b^	17.26^a^	0.74	0.44	< 0.001	0.79
Blood flow, % of total	81.5	83.7	2.1	84.5^a^	83.7^a^	83.0^ab^	82.2^bc^	80.9^c^	81.4^c^	1.8	0.47	0.006	0.76
*Contralateral uterine artery*													
Peak systolic velocity, cm/s	205	193	17	156^d^	177^c^	203^b^	207^ab^	221^a^	229^a^	19	0.59	< 0.001	0.97
End diastolic velocity, cm/s	88	81	8	63^d^	75^c^	88^b^	90^ab^	96^a^	97^ab^	9	0.52	< 0.001	0.92
Mean velocity, cm/s	137	125	12	96^d^	114^c^	134^b^	139^b^	149^a^	155^a^	13	0.48	< 0.001	0.91
Pulsatility index	0.866	0.892	0.031	1.002^a^	0.891^b^	0.854^bc^	0.839^c^	0.839^c^	0.852^bc^	0.044	0.54	< 0.001	0.86
Resistance index	0.568	0.573	0.011	0.598^a^	0.570^b^	0.559^b^	0.561^b^	0.562^b^	0.573^b^	0.014	0.74	0.01	0.82
Cross-sectional area, cm^2^	0.309	0.274	0.022	0.147^f^	0.200^e^	0.258^d^	0.320^c^	0.389^b^	0.432^a^	0.027	0.26	< 0.001	0.65
Blood flow, L/min	2.76	2.28	0.34	0.87^f^	1.42^e^	2.14^d^	2.85^c^	3.62^b^	4.21^a^	0.45	0.32	< 0.001	0.62
Total blood flow, L/min	14.16	13.05	0.63	5.56^f^	8.51^e^	12.35^d^	15.24^c^	18.49^b^	21.48^a^	0.82	0.20	< 0.001	0.73
Total blood flow relative to calf birth weight^4^, mL·min^−1^·kg^−1^	472	437	30	184^f^	283^e^	414^d^	513^c^	615^b^	717^a^	33	0.39	< 0.001	0.66
Total blood flow relative to dam BW^5^, mL·min^−1^·kg^−1^	29.3	28.9	1.4	12.3^f^	18.6^e^	26.7^d^	32.4^c^	39.5^b^	45.0^a^	1.7	0.83	< 0.001	0.92

^a,b,c,d,e,f^Means differ (*P* ≤ 0.05) for main effect of day.

^1^Primiparous dams were individually-fed either 100% (Control; CON) or 70% (Nutrient Restricted; NR) of estimated metabolizable energy and metabolizable protein requirements for maintenance, pregnancy, and growth from day 160 of gestation to parturition.

^2^Probabilities of difference for late gestational nutritional plane (Nutr), day of gestation (Day), and their interaction.

^3^Standard error of the mean for CON (*n* = 10 or 11) and NR (*n* = 13).

^4^Calf birth weight determined at 0.9 ± 0.3 h (SD) of age (pre-suckling).

^5^Preprandial dam body weight (BW) determined at each respective timepoint.

Contralateral uterine artery peak systolic velocity, end diastolic velocity, and mean velocity increased (*P* ≤ 0.005; Table 1) from days 157 to 202 of gestation. Additionally, mean velocity of the contralateral uterine artery increased (*P* = 0.05) and peak systolic velocity and end diastolic velocity tended to increase (*P* ≤ 0.10) from days 223 to 244 of gestation. Pulsatility and resistance indices of the contralateral uterine artery decreased (*P* ≤ 0.01) from days 157 to 181, and pulsatility index tended to decrease (*P* = 0.07) from days 181 to 202 of gestation. From days 157 to 265 of gestation, contralateral uterine artery cross-sectional area and blood flow increased (*P* ≤ 0.003; Table 1; Figure 4).

Total uterine artery blood flow, total blood flow relative to calf birth weight, and total blood flow relative to dam BW increased (*P* ≤ 0.001; Table 1; Figure 4) from days 157 to 265 of gestation.

### Placental size

Ipsilateral dry cotyledonary, intercotyledonary, and placental weights were not affected (*P* ≥ 0.38; Table 2) by late gestational nutritional plane. Additionally, nutritional plane did not affect (*P* ≥ 0.19) ipsilateral number of cotyledons and average dry cotyledon weight. Ipsilateral dry cotyledonary:intercotyledonary weight was less (*P* = 0.02) for NR dams than CON. As a percentage of total weight or number, ipsilateral dry intercotyledonary and placental weight were greater (*P* ≤ 0.05) and ipsilateral cotyledonary weight and number of cotyledons tended to be greater (*P* ≤ 0.08) for NR dams than CON.

**Table 2.** Effects of late gestational nutritional plane of primiparous beef females on placental size

	Nutritional plane^1^			
Item	CON	NR	SEM^2^	*P*-value
*Ipsilateral side*				
Dry cotyledonary weight, g	69.0	67.3	4.1	0.77
Dry intercotyledonary weight, g	103	110	6	0.38
Dry placental weight, g	172	177	10	0.68
Number of cotyledons	50.0	53.1	2.3	0.35
Average dry cotyledon weight, g	1.39	1.24	0.08	0.19
Dry cotyledonary:intercotyledonary weight	0.676	0.610	0.018	0.02
Dry cotyledonary weight^3^, % of total	78.2	84.8	2.5	0.08
Dry intercotyledonary weight^3^, % of total	81.8	87.1	1.8	0.05
Dry placental weight^3^, % of total	80.1	86.2	1.9	0.03
Number of cotyledons^3^, % of total	55.6	64.8	3.4	0.07
*Contralateral side*				
Dry cotyledonary weight, g	18.7	11.8	2.3	0.04
Dry intercotyledonary weight, g	22.2	15.9	2.4	0.08
Dry placental weight, g	40.9	27.7	4.1	0.03
Number of cotyledons	40.4	32.2	3.4	0.10
Average dry cotyledon weight, g	0.452	0.342	0.047	0.12
Dry cotyledonary:intercotyledonary weight	0.924	0.755	0.134	0.37
*Whole placenta*				
Dry cotyledonary weight, g	87.0	77.1	4.5	0.13
Dry intercotyledonary weight, g	125	129	7	0.72
Dry total placental weight, g	211	204	11	0.64
Number of cotyledons	90.7	83.8	3.7	0.20
Average dry cotyledon weight, g	0.975	0.935	0.057	0.63
Dry cotyledonary:intercotyledonary weight	0.711	0.613	0.026	0.01
Placental efficiency^4^, kg calf birth weight/g total placental DM	0.149	0.151	0.006	0.89
Placental weight relative to dam BW^5^, g total placental DM/kg dam BW	0.438	0.493	0.023	0.09
Average umbilical vessel diameter, mm	7.35	7.68	0.18	0.21

^1^Primiparous dams were individually-fed either 100% (Control; CON) or 70% (Nutrient Restricted; NR) of estimated metabolizable energy and metabolizable protein requirements for maintenance, pregnancy, and growth from day 160 of gestation to parturition.

^2^Standard error of the mean for CON (*n* = 11) and NR (*n* = 11 or 12).

^3^Ipsilateral side dry weight as a percentage of whole placenta dry weight.

^4^Calf birth weight determined at 0.9 ± 0.3 h (SD) of age (pre-suckling).

^5^Dam body weight (BW) determined post-calving (days 1 and 2 of lactation).

Contralateral dry cotyledonary weight was less (*P* = 0.04; Table 2) and intercotyledonary weight tended to be less (*P* = 0.08) for NR dams than CON. This resulted in contralateral dry placental weight being less (*P* = 0.03) for NR dams than CON. Contralateral number of cotyledons tended to be less (*P* = 0.10) for NR dams than CON, but average dry cotyledon weight and cotyledonary:intercotyledonary weight were not affected (*P* ≥ 0.12) by late gestational nutritional plane.

Late gestational nutritional plane did not affect (*P* ≥ 0.13; Table 2) whole dry cotyledonary, intercotyledonary, and total placental weights or the number of cotyledons and average dry cotyledon weight. Whole placental dry cotyledonary:intercotyledonary weight was less (*P* = 0.01) for NR dams than CON. Placental efficiency and average umbilical vessel diameter were not affected (*P* ≥ 0.21) by nutritional plane, but whole placental weight relative to dam BW tended to be greater (*P* = 0.09) for NR dams than CON.

### Cotyledonary relative mRNA expression

Cotyledonary relative mRNA expression of GLUT3 and SNAT2 was greater (*P* ≤ 0.05; Table 3) and relative expression of GLUT1 and GLUT4 tended to be greater (*P* ≤ 0.07) for NR dams than CON. Late gestational nutritional plane did not affect (*P* ≥ 0.31) cotyledonary relative mRNA expression of GLUT5, 4F2hc, CAT1, LAT1, and LAT2. Nutrient restricted dams tended to have greater (*P* = 0.07) cotyledonary relative mRNA expression of NOS3, but relative expression of VEGFA, FLT1, KDR, GUCY1B3, and PAG2 was not affected (*P* ≥ 0.13) by nutritional plane.

**Table 3.** Effects of late gestational nutritional plane of primiparous beef females on cotyledonary relative mRNA expression^1^

	Nutritional plane^2^			
Item	CON	NR	SEM^3^	*P*-value
*Glucose transporters*				
GLUT1 (SLC2A1)	0.72	0.90	0.07	0.07
GLUT3 (SLC2A3)	0.71	0.94	0.07	0.02
GLUT4 (SLC2A4)	0.77	1.22	0.17	0.06
GLUT5 (SLC2A5)	0.81	0.83	0.11	0.89
*Amino acid transporters*				
4F2hc (SLC3A2)	1.03	1.09	0.09	0.61
CAT1 (SLC7A1)	0.82	1.01	0.14	0.31
LAT1 (SLC7A5)	1.15	1.08	0.14	0.74
LAT2 (SLC7A8)	1.14	1.11	0.10	0.82
SNAT2 (SLC38A2)	1.00	1.32	0.11	0.05
*Angiogenic factors*				
VEGFA	0.99	1.03	0.11	0.78
FLT1 (VEGFA receptor 1)	1.04	1.34	0.14	0.13
KDR (VEGFA receptor 2)	1.04	1.32	0.18	0.30
NOS3	0.74	1.07	0.12	0.07
GUCY1B3 (nitric oxide receptor)	0.83	1.02	0.10	0.15
PAG2	0.75	0.78	0.12	0.90

^1^Target mRNA cycle threshold values were normalized to G3PDH (reference gene) with mRNA expression calculated relative to a pooled internal control using the 2^−ΔΔCT^ method.

^2^Primiparous dams were individually-fed either 100% (Control; CON) or 70% (Nutrient Restricted; NR) of estimated metabolizable energy and metabolizable protein requirements for maintenance, pregnancy, and growth from day 160 of gestation to parturition.

^3^Standard error of the mean for CON (*n* = 11 or 12) and NR (*n* = 11).

## Discussion

We previously reported that during late gestation, control dams gained maternal (non-gravid) BW, maintained BCS and backfat thickness, and were metabolically stable (Redifer et al., 2023). Conversely, nutrient restricted dams decreased maternal BW, BCS, and backfat thickness, had elevated NEFA concentrations, and had reduced circulating glucose, urea N, and triglycerides during late gestation. Although nutrient restricted dams weighed 64 kg less and were 2.0 BCS lower than controls post-calving, calf BW and size at birth were not affected (Redifer et al., 2023). Overall, data from the current study suggest that first-parity beef females that were nutrient restricted during late gestation maintained total uterine artery blood flow, had similar total placental mass, and made placental adaptations, which collectively allowed for fetal growth to be spared.

### Uterine artery blood flow

Pregnancy results in considerable systemic cardiovascular adaptation including decreases in arterial blood pressure and systemic vascular resistance along with increases in cardiac output, stroke volume, heart rate, and blood volume (Robson et al., 1989). In late gestation, approximately half of the increased cardiac output is directed to the gravid uterus and mammary gland (Rosenfeld, 1977). Color Doppler ultrasonography provides a non-invasive, repeatable method to measure changes in uterine artery blood flow throughout the bovine pregnancy (Bollwein et al., 2002; Panarace et al., 2006). Contrary to our hypothesis, first-parity females that were nutrient restricted during late gestation were able to adapt to undernutrition and prioritize blood flow to the gravid uterus, as uterine artery hemodynamics and total blood flow were not affected. However, maternal heart rate decreased for nutrient restricted dams from days 157 to 181 of gestation and remained between 11 and 13% less than controls for the rest of pregnancy.

During the latter half of pregnancy, inter-individual variability in total uterine blood flow has been reported to be substantial in dairy cows (Herzog et al., 2011). To minimize variation in the current study, single-sired females were bred to a single sire, the angle of insonation was kept in a fairly narrow range and maintained consistently within a female, and 1 trained technician conducted all scans during a similar time frame each day. Still, total uterine blood flow CV ranged between 17 and 21% across females within each timepoint. By side, the ipsilateral CV ranged between 20 and 25%, while the contralateral CV ranged between 37 and 57%. Along with the data presented here, additional ultrasound scans were conducted on days 104 and 138 of gestation, but were not included in the repeated measures analysis. Total uterine blood flow did not differ between nutritional planes on days 104 (*P* = 0.88; 1.65 vs. 1.67 ± 0.13 L/min, for control vs. nutrient restricted) and 138 of gestation (*P* = 0.95; 4.19 vs. 4.17 ± 0.29 L/min). Knowing that uterine blood flow was similar prior to treatment initiation and measuring blood flow at 5 time points during late gestation gives us confidence that uterine artery blood flow was not affected by nutrient restriction in this study.

In fall-calving heifers that were nutrient restricted during late gestation, uterine blood flow on day 220 of gestation was not affected; however, in the spring-calving cohort of the same study, nutrient restriction decreased ipsilateral and total uterine blood flow (Contreras-Correa et al., 2021). Despite nutrient restricted heifers gaining maternal BW during late gestation, fetal weight on day 240 of gestation was less than for controls in both calving seasons (Contreras-Correa et al., 2021). Mature beef cows fed poor-quality forage during late gestation had decreased ipsilateral and total uterine blood flow, and smaller calf birth weight compared with cows supplemented with DDGS (Kennedy et al., 2016). Interestingly, in a similar study from the same laboratory, DDGS-supplemented dams had decreased ipsilateral and total uterine blood flow, but calf birth weight was not affected (Mordhorst et al., 2017). Supplementing poor-quality forage diets with corn during mid- and late gestation did not affect uterine blood flow or calf birth weight in mature beef cows (Tanner et al., 2023). Heifers that were nutrient restricted during mid-gestation had decreased ipsilateral and total uterine blood flow (Lemley et al., 2018). Uterine blood flow was not determined pre-treatment in most of the studies above (Mordhorst et al., 2017; Lemley et al., 2018; Contreras-Correa et al., 2021), so it cannot be ruled out that blood flow was divergent before study initiation. Clearly, the effect of gestational nutritional status on uterine blood flow in beef cattle is still poorly understood, and little has been postulated to explain the variation in outcomes due to poor nutrition or the physiological mechanisms responsible.

In dairy cows, changes in maternal heart rate followed changes in energy intake (Thomas and Moore, 1951). We previously reported that upon being re-fed to meet estimated nutrient requirements during lactation, nutrient restricted dams from the current study had greater maternal heart rate than controls (Redifer et al., 2024b). Together, these results support the hypothesis of Thomas and Moore (1951) that heart rate is related to changes in nutritional plane. In agreement, mature beef cows fed poor-quality forage had lower maternal heart rate than cows supplemented with DDGS (Kennedy et al., 2016). Other studies supplementing poor-quality forage diets resulted in minimal divergence in cow performance (Mordhorst et al., 2017; Tanner et al., 2023), which likely explains the lack of effect on maternal heart rate. In Lemley et al. (2018), heifer BW was 70 kg less after mid-gestational undernutrition, so it is surprising that maternal heart rate was not decreased in accordance.

Bollwein et al. (2002) and Panarace et al. (2006) described changes in uterine hemodynamics throughout pregnancy, where flow velocities, vessel cross-sectional area, and blood flow increased, while pulsatility and resistance indices decreased in both uterine arteries. Furthermore, these authors demonstrated that the ipsilateral uterine artery had greater flow velocities, vessel cross-sectional area, and blood flow, but lower pulsatility and resistance indices than the contralateral uterine artery throughout late gestation (Bollwein et al., 2002; Panarace et al., 2006), in agreement with our data. While variation exists in absolute values for uterine hemodynamics across studies, likely due to maternal (parity, BW, and breed), fetoplacental (genetic potential for growth, fetal sex), environmental (temperature, season), and methodological (angle of insonation, location on the uterine artery) factors, trends over time are comparable with the current study.

From days 157 to 265 of gestation, ipsilateral and contralateral blood flow increased 2.7- and 3.8-fold, respectively. Uterine arterio-venous concentration differences change minimally during pregnancy (Reynolds et al., 1986), but increasing blood flow allows transplacental uptake to keep pace with the nutrient demands of the rapidly-growing fetus. The increase in blood flow of both uterine arteries through day 265 of gestation was largely driven by continual growth in vessel cross-sectional area, more so than the few increases in flow velocities early during nutritional treatments.

Pulsatility and resistance indices decreased early in the third trimester and remained generally static for the rest of late gestation. The decrease in these indices is indicative of a low resistance system due to the uteroplacental vascular remodeling that occurs during pregnancy (Osol and Mandala, 2009). In humans, if vascular resistance remains persistently elevated and does not normalize until late in pregnancy, there is a greater likelihood of adverse perinatal outcomes (Kurdi et al., 2004; Gómez et al., 2006). In the current study, resistance index of the ipsilateral uterine artery was numerically greatest on day 265 of gestation, but uterine vascular resistance had clearly normalized relative to early pregnancy values, and likely was not biologically concerning. After the initial divergence in maternal heart rate due to nutritional plane, heart rate increased from days 202 to 223 and days 244 to 265 for dams on both nutritional planes, partially accounting for increased cardiac output during pregnancy.

For the ipsilateral uterine artery, flow velocities, and pulsatility and resistance indices plateaued by days 202 and 181 of gestation, respectively. The contralateral uterine artery had similar changes early in the third trimester, but pulsatility index tended to decrease again from days 181 to 202 of gestation and mean velocity increased from days 223 to 244 of gestation. It appears that final vascular remodeling is completed earlier for the ipsilateral horn relative to the contralateral horn. The continued vascular adaptations later in pregnancy of the contralateral artery are likely responsible mechanisms for contralateral blood flow representing a greater proportion of total blood flow from days 223 to 265 of gestation compared with days 157 and 181 of gestation.

### Placental size

Bovine placental growth increases exponentially throughout pregnancy (Reynolds et al., 1990). In fact, placental growth is preceded by increased uterine blood flow (Reynolds and Ferrell, 1987) and is more advanced than fetal growth on any day of pregnancy (Ferrell et al., 1976; Prior and Laster, 1979) to ensure sufficient placental nutrient transport capacity. The current study is the first known report to separate bovine placentas into ipsilateral and contralateral sides. In our study, first-parity beef females that were nutrient restricted during late gestation had lower cotyledonary relative to intercotyledonary tissue, less contralateral placental growth, and a greater ipsilateral side relative to the total placenta than control dams. Despite placental adaptations within sides, whole placental weight and placental efficiency were not affected by late gestational nutritional plane.

We previously reported moderate positive correlations among late gestational total uterine artery blood flow, dry total placental weight, and calf birth weight (Redifer and Meyer, 2020; Redifer et al., 2021). Mechanistically, it is still unclear if uterine blood flow drives placental and fetal growth or if fetoplacental growth potential and endocrine signaling dictate uterine blood flow changes. In the current study, calf birth weight of the subset of dams for which expelled placentas were collected successfully was not affected (*P* = 0.64; 30.2 vs. 31.2 ± 1.2) by late gestational nutritional plane, as was true for the entire population. While our hypothesis that whole placental weight would be decreased was rejected, it is logical that uterine blood flow, placental weight, and calf birth weight were similarly unchanged.

In agreement with the current study, late gestational energy restriction in heifers did not affect placentome weight, placentome number, or fetal weight (Long et al., 2021). Expelled placental weights were not affected by poor maternal nutrition during mid- and late (Tanner et al., 2023) or late (Kennedy et al., 2016; Mordhorst et al., 2017; Hummel et al., 2021) gestation in mature beef cows, with lower calf birth weights observed only by Kennedy et al. (2016). Placental size appears to often be resilient to late gestational undernutrition in beef females, regardless of fetal growth outcomes, suggesting other factors of placental nutrient transport capacity are likely involved.

Our laboratory previously categorized beef dams by peripartum BCS category, and thin (<5.00) dams had lower placental dry weights than moderate (5.00 to 5.99) and fleshy (≥6.00) dams, while birth weight was less for thin and moderate dams compared with fleshy dams (Redifer et al., 2021). Alternatively, when mature beef cows were fed to achieve a BCS of 4.0 or 6.0 during mid-gestation and maintained at that BCS until day 259 of gestation, BCS 4.0 dams had greater placental weights, but there was no effect on fetal weight (Rasby et al., 1991). Past studies largely reported placental wet weights, but we demonstrated that after rinsing expelled placentas to remove contamination, DM determination is beneficial to eliminate variation (Redifer et al., 2021). Corroborating this notion, Perry et al. (1999) did not observe a treatment difference for wet cotyledon weight, but dry cotyledon weight was less due to early gestational protein restriction.

It has been previously postulated that greater uterine blood flow to the ipsilateral horn is related to greater placental mass located in that horn, and changes in uterine blood flow are accompanied by similar changes in placental growth on a respective side (Bollwein et al., 2002; Camacho et al., 2014), but this has not been demonstrated in cattle. By separating placentas into ipsilateral and contralateral sides, we confirmed that along with substantially greater ipsilateral uterine blood flow, the ipsilateral uterine horn contains considerably more placental tissue. Ipsilateral blood flow and placental weights as a proportion of the totals were in a similar range. These observations support the idea that evaluating uterine blood flow and placental size by side can provide insight into the contribution of each horn of the gravid uterus to a successful pregnancy. Further data using this approach can help to determine if placental insufficiency and intrauterine growth restriction may be due to side-specific alterations.

A reduction in the number of contralateral cotyledons for nutrient restricted dams was an unexpected finding in the current study. Small cotyledons (>20 mm in diameter) were not dissected from intercotyledonary tissue or included in cotyledon number, but small cotyledons were noted for the contralateral side of 2 nutrient restricted dams. The number of functional placentomes is thought to be established near the end of the first trimester in the bovine pregnancy (Laven and Peters, 2001; Assis Neto et al., 2010). With its number set, placentomal growth adapts during late gestation to facilitate nutrient demands of fetal growth, and perhaps undernutrition resulted in poor placentomal development and greater occurrence of small cotyledons in nutrient restricted dams. Both ipsilateral and contralateral blood flow numerically separated towards the end of pregnancy, being less in nutrient restricted dams, but only contralateral placental size was less at term. This suggests that ipsilateral placental growth is prioritized over contralateral placental growth, likely because of local factors released by the growing fetus located on that side. Still, despite contralateral placental growth not being able to keep up in nutrient restricted dams, other placental adaptations seem to have occurred so that fetal growth was spared.

### Cotyledonary relative mRNA expression

Placental nutrient transport capacity is influenced by the abundance, affinity, and localization of nutrient transporters in the placenta (Fowden et al., 2006). In the epitheliochorial placenta of ruminants, maternally-derived nutrients must traverse both the apical and basal membranes of the caruncle as well as the cotyledon to reach fetal circulation (Peter, 2013). Often, pregnant dams are slaughtered or cesarean-sections are performed to sample both cotyledonary and caruncular tissues when nutrient transport is actively occurring, but that prevents the birth of viable calves. Conversely, the current experimental design resulted in full-term calves for further research objectives (Wichman et al., 2023; Redifer et al., 2024a), but only cotyledonary tissue could be sampled from naturally-expelled placentas. Picking et al. (2020) sampled placentomes from 4 locations within the ipsilateral and contralateral sides and reported no effect of location on expression of nutrient transporters and angiogenic factors, suggesting that our results from the center of the ipsilateral side can be extrapolated to cotyledons of the entire placenta.

Glucose concentrations are greater in maternal circulation than fetal circulation (Ferrell et al., 1983); thus, glucose is transferred down the maternal-fetal gradient by facilitated diffusion (Widdas, 1952) to be used as the primary source of metabolic fuel for the fetus and placenta (Hay, 2006). In our study, late gestational nutrient restriction resulted in greater cotyledonary mRNA expression of the glucose transporters (GLUT1, GLUT3, and GLUT4), but not the fructose transporter (GLUT5). Nutrient restricted dams had less circulating glucose during late gestation and at 1 h post-calving (Redifer et al., 2023), yet neonatal calf pre-suckling serum glucose concentrations were not affected by late gestational nutritional plane (Wichman et al., 2023). Upregulation of glucose transporters likely aided in ensuring available energy substrates were adequate, contributing to spared fetal and placental growth.

Previously, late gestational undernutrition resulted in greater cotyledonary mRNA expression of GLUT8 in spring-calving heifers (Swanson et al., 2022), but the major glucose transporters were not measured, and fetal weight was still compromised (Contreras-Correa et al., 2021). Glucose transporter expression was not affected by nutrient restriction in the fall-calving contemporaries of Swanson et al. (2022) or by Picking et al. (2020). In contrast to our findings, decreasing diet energy density for the last month of gestation resulted in smaller calf birth weight and lower expelled cotyledonary expression of GLUT1 and GLUT3 (Kang et al., 2022). It is surprising that such a minimal change in dietary energy density (8.76 vs. 9.47 vs. 10.18 MJ/kg) for a short duration was able to alter placental gene expression and fetal growth by Kang et al. (2022), but nutrient intakes and maternal performance responses were not sufficiently described.

During pregnancy, fetal amino acid concentrations are greater than maternal concentrations (Ferrell et al., 1983), and amino acids are transported against a fetal-maternal concentration gradient involving energy-dependent, active transport (Young and Fadyen, 1973). Amino acids are used for tissue growth and as oxidative fuel by the fetus, but there is extensive metabolism that occurs within the placenta altering the profile of amino acids available (Battaglia and Regnault, 2001). We observed that late gestational nutrient restriction increased cotyledonary mRNA expression of a system A neutral amino acid transporter (SNAT2) but did not alter mRNA expression of system L neutral amino acid transporters (4F2hc, LAT1, and LAT2) or a cationic amino acid transporter (CAT1). Much like the greater expression of glucose transporters, increased SNAT2 expression in nutrient restricted dams was likely a compensatory mechanism to improve the efficiency of nutrient uptake for fetal and placental growth. Expression of amino acid transporters was not affected by late gestational undernutrition for cotyledons in Swanson et al. (2022) or placentomes in Picking et al. (2020). Low dietary energy density for the last month of pregnancy resulted in less SNAT1 transcripts but did not affect SNAT2 or SNAT4 transcripts compared with high dietary energy density (Kang et al., 2022).

Placental angiogenesis, or the formation of new blood vessels from the existing vasculature, is a prerequisite for tissue growth and development and is regulated in part by vascular endothelial growth factor A and nitric oxide (Reynolds et al., 2010a). First-parity females that were nutrient restricted during late gestation had increased cotyledonary mRNA expression of NOS3, but the receptor for nitric oxide (GUCY1B3), and VEGFA along with its receptors (FLT1 and KDR) were not affected in the current study. Previously, late gestational undernutrition in beef cattle did not affect placentomal VEGFA and FLT1 transcripts (Picking et al., 2020) but decreased cotyledonary VEGFA and NOS3 transcripts (Contreras-Correa et al., 2022). Placentomes collected from a mid- and late gestational nutrient restriction model had decreased enrichment of pathways associated with vascular, cardiovascular, and circulatory systems, but increased enrichment of pathways associated with translation and ribosomal function (Reid et al., 2022). Lemley et al. (2018) reported no effect of mid-gestational nutrient restriction on angiogenic factor abundance but observed positive relationships between macroscopic blood vessel density and cotyledonary expression of VEGF, FLT1, and KDR. While cotyledonary vascularity was not measured in the current study, we cannot eliminate the possibility that histological differences existed in nutrient restricted dams as an additional compensatory response.

Bovine pregnancy-associated glycoproteins are produced by the placenta in increasing quantities as gestation progresses, with PAG2 localized in mononucleated and binucleated cotyledonary trophoblast (Wallace et al., 2015). While the role of pregnancy-associated glycoproteins is still not completely clear, they have been associated with immunomodulatory activity, luteotropic function, adhesive capacity, and proteolytic ability (Wallace et al., 2015). Late gestational nutrient restriction previously had no effect on placentomal mRNA expression of PAG1 (Picking et al., 2020), which concurs with our current findings for PAG2.

## Conclusions

In summary, these data illustrate the resiliency of the gravid uteroplacenta to preserve placental nutrient transport capacity when first-parity beef females were nutrient restricted during late gestation. Undernutrition was detrimental to maternal growth during late pregnancy, and as a result, nutrient restricted dams had less maternal BW and BCS than controls post-calving (Redifer et al., 2023). Despite impaired maternal growth and energy reserves, undernutrition resulted in minimal negative effects and even some compensatory adaptations by the uteroplacenta. Maternal heart rate was less for nutrient restricted dams, but total uterine artery blood flow was maintained, and uterine hemodynamics were not affected. Placentas of nutrient restricted dams had less cotyledonary tissue relative to intercotyledonary tissue and a smaller contralateral side, but compensatory responses included greater expression of 4 nutrient transporters and 1 angiogenic factor, and whole placental mass was not affected. Overall, these results suggest that placental nutrient transport capacity was not severely altered by late gestational nutrient restriction in first-parity beef heifers, allowing for fetal growth to be spared (Redifer et al., 2023). Given that the dam is not always able to withstand late gestational nutrient restriction without compromising fetal growth, continued investigation into the underlying mechanisms behind contrasting uteroplacental responses to late gestational undernutrition is warranted.

## Abbreviations

ADF: acid detergent fiber
BCS: body condition score
BW: body weight
cDNA: complimentary deoxyribonucleic acid
CP: crude protein
DM: dry matter
DDGS: dried distillers’ grains with solubles
ME: metabolizable energy
MP: metabolizable protein
mRNA: messenger ribonucleic acid
NDF: neutral detergent fiber
NR: nutrient restricted
qRT-PCR: quantitative real-time polymerase chain reaction
RNA: ribonucleic acid

## References

[CIT0001] Assis Neto, A. C., F. T. V. Pereira, T. C. Santos, C. E. Ambrosio, R. Leiser, and M. A. Miglino. 2010. Morpho‐physical recording of bovine conceptus (Bos indicus) and placenta from days 20 to 70 of pregnancy. Reprod. Dom. Anim. 45:760–772. doi: 10.1111/j.1439-0531.2009.01345.x 19281595

[CIT0002] Battaglia, F., and T. Regnault. 2001. Placental transport and metabolism of amino acids. Placenta 22:145–161. doi: 10.1053/plac.2000.0612 11170819

[CIT0003] Bollwein, H., U. Baumgartner, and R. Stolla. 2002. Transrectal Doppler sonography of uterine blood flow in cows during pregnancy. Theriogenology. 57:2053–2061. doi: 10.1016/s0093-691x(02)00706-9 12066865

[CIT0004] Camacho, L. E., C. O. Lemley, L. D. Prezotto, M. L. Bauer, H. C. Freetly, K. C. Swanson, and K. A. Vonnahme. 2014. Effects of maternal nutrient restriction followed by realimentation during midgestation on uterine blood flow in beef cows. Theriogenology. 81:1248–1256. doi: 10.1016/j.theriogenology.2014.02.006 24650930

[CIT0005] Carter, A. M. 2012. Evolution of placental function in mammals: the molecular basis of gas and nutrient transfer, hormone secretion, and immune responses. Physiol. Rev. 92:1543–1576. doi: 10.1152/physrev.00040.2011 23073626

[CIT0007] Contreras-Correa, Z. E., R. D. Messman, D. R. Sidelinger, E. Heath King, H. L. Sánchez-Rodríguez, D. D. Burnett, and C. O. Lemley. 2021. Melatonin alters bovine uterine artery hemodynamics, vaginal temperatures, and fetal morphometrics during late gestational nutrient restriction in a season-dependent manner. J. Anim. Sci. 99:skab242. doi: 10.1093/jas/skab242 34387666 PMC8420683

[CIT0006] Contreras-Correa, Z. E., T. Cochran, A. Metcalfe, D. D. Burnett, and C. O. Lemley. 2022. Seasonal and temporal variation in the placenta during melatonin supplementation in a bovine compromised pregnancy model. J. Anim. Sci. 100:skac372. doi: 10.1093/jas/skac372 36370127 PMC9762882

[CIT0008] Dyce, K. M., and C. J. G. Wensing. 1971. Essentials of bovine anatomy. Philadelphia, PA: Lea & Febiger.

[CIT0010] Ferrell, C. L., W. N. Garrett, and N. Hinman. 1976. Growth, development and composition of the udder and gravid uterus of beef heifers during pregnancy. J. Anim. Sci. 42:1477–1489. doi: 10.2527/jas1976.4261477x 931823

[CIT0009] Ferrell, C. L., S. P. Ford, R. L. Prior, and R. K. Christenson. 1983. Blood flow, steroid secretion and nutrient uptake of the gravid bovine uterus and fetus. J. Anim. Sci. 56:656–667. doi: 10.2527/jas1983.563656x 6841301

[CIT0011] Fowden, A. L., J. W. Ward, F. P. B. Wooding, A. J. Forhead, and M. Constancia. 2006. Programming placental nutrient transport capacity. J. Physiol. 572:5–15. doi: 10.1113/jphysiol.2005.104141 16439433 PMC1779642

[CIT0012] Freetly, H. C., C. L. Ferrell, and T. G. Jenkins. 2005. Nutritionally altering weight gain patterns of pregnant heifers and young cows changes the time that feed resources are offered without any differences in production. J. Anim. Sci. 83:916–926. doi: 10.2527/2005.834916x 15753348

[CIT0013] Getty, R. 1964. Atlas for applied veterinary anatomy. 2nd ed. Ames, IA: Iowa State University Press.

[CIT0014] Gómez, O., F. Figueras, J. M. Martínez, M. Río, M. Palacio, E. Eixarch, B. Puerto, O. Coll, V. Cararach, and J. A. Vanrell. 2006. Sequential changes in uterine artery blood flow pattern between the first and second trimesters of gestation in relation to pregnancy outcome. Ultrasound Obstet. Gynecol. 28:802–808. doi: 10.1002/uog.2814 17063456

[CIT0015] Hay, W. W. Jr . 2006. Placental-fetal glucose exchange and fetal glucose metabolism. Trans. Am. Clin. Climatol. Assoc. 117:321–39. 18528484 PMC1500912

[CIT0016] Herzog, K., J. Koerte, G. Flachowsky, and H. Bollwein. 2011. Variability of uterine blood flow in lactating cows during the second half of gestation. Theriogenology. 75:1688–1694. doi: 10.1016/j.theriogenology.2010.12.033 21334053

[CIT0017] Holland, M. D., and K. G. Odde. 1992. Factors affecting calf birth weight: a review. Theriogenology. 38:769–798. doi: 10.1016/0093-691x(92)90155-k 16727179

[CIT0018] Hummel, G., K. Woodruff, K. Austin, R. Knuth, S. Lake, and H. Cunningham-Hollinger. 2021. Late gestation maternal feed restriction decreases microbial diversity of the placenta while mineral supplementation improves richness of the fetal gut microbiome in cattle. Animals. 11:2219. doi: 10.3390/ani11082219 34438676 PMC8388467

[CIT0019] Kang, K., L. Zeng, J. Ma, L. Shi, R. Hu, H. Zou, Q. Peng, L. Wang, B. Xue, and Z. Wang. 2022. High energy diet of beef cows during gestation promoted growth performance of calves by improving placental nutrients transport. Front. Vet. Sci. 9:1053730. doi: 10.3389/fvets.2022.1053730 36504847 PMC9730878

[CIT0020] Kennedy, V. C., B. R. Mordhorst, J. J. Gaspers, M. L. Bauer, K. C. Swanson, C. O. Lemley, and K. A. Vonnahme. 2016. Supplementation of corn dried distillers’ grains plus solubles to gestating beef cows fed low-quality forage: II. Impacts on uterine blood flow, circulating estradiol-17β and progesterone, and hepatic steroid metabolizing enzyme activity. J. Anim. Sci. 94:4619–4628. doi: 10.2527/jas.2016-0400 27898957

[CIT0021] Kurdi, W., A. Fayyad, V. Thakur, and K. Harrington. 2004. Delayed normalization of uterine artery Doppler waveforms is not a benign phenomenon. Eur. J. Obstet. Gynecol. Reprod. Biol. 117:20–23. doi: 10.1016/j.ejogrb.2003.10.038 15474238

[CIT0022] Laven, R. A., and A. R. Peters. 2001. Gross morphometry of the bovine placentome during gestation. Reprod. Domest. Anim. 36:289–296. doi: 10.1046/j.1439-0531.2001.00297.x 11928923

[CIT0023] Lemley, C. O., C. G. Hart, R. L. Lemire, E. H. King, R. M. Hopper, S. B. Park, B. J. Rude, and D. D. Burnett. 2018. Maternal nutrient restriction alters uterine artery hemodynamics and placentome vascular density in Bos indicus and Bos taurus. J. Anim. Sci. 96:4823–4834. doi: 10.1093/jas/sky329 30107547 PMC6247851

[CIT0024] Livak, K. J., and T. D. Schmittgen. 2001. Analysis of relative gene expression data using real-time quantitative PCR and the 2^-ΔΔCT^ Method. Methods 25:402–408. doi: 10.1006/meth.2001.1262 11846609

[CIT0025] Long, J. M., L. A. Trubenbach, J. H. Pryor, C. R. Long, T. A. Wickersham, J. E. Sawyer, and M. C. Satterfield. 2021. Maternal nutrient restriction alters endocrine pancreas development in fetal heifers. Domest Anim. Endocrinol. 74:106580. doi: 10.1016/j.domaniend.2020.106580 33160154

[CIT0026] May, N. D. S. 1970. The anatomy of the sheep. 3rd ed. St. Lucia, Queensland: University of Queensland Press.

[CIT0027] Meyer, A. M., and C. A. Redifer. 2024. The curse of the firstborn: effects of dam primiparity on developmental programming in ruminant offspring. Anim. Reprod. Sci. 265:107469. doi: 10.1016/j.anireprosci.2024.107469 38705081

[CIT0028] Mordhorst, B. R., C. A. Zimprich, L. E. Camacho, M. L. Bauer, and K. A. Vonnahme. 2017. Supplementation of distiller’s grains during late gestation in beef cows consuming low‐quality forage decreases uterine, but not mammary, blood flow. J. Anim. Physiol. Anim. Nutr. 101:e154–e164. doi: 10.1111/jpn.12580 27874218

[CIT0029] NASEM. 2016. Nutrient requirements of beef cattle. 8th rev. ed. Washington, DC: National Academic Press. doi: 10.17226/19014

[CIT0030] Osol, G., and M. Mandala. 2009. Maternal uterine vascular remodeling during pregnancy. Physiology (Bethesda). 24:58–71. doi: 10.1152/physiol.00033.2008 19196652 PMC2760472

[CIT0031] Panarace, M., C. Garnil, M. Marfil, G. Jauregui, J. Lagioia, E. Luther, and M. Medina. 2006. Transrectal Doppler sonography for evaluation of uterine blood flow throughout pregnancy in 13 cows. Theriogenology. 66:2113–2119. doi: 10.1016/j.theriogenology.2006.03.040 16876855

[CIT0032] Perry, V. E. A., S. T. Norman, J. A. Owen, R. C. Daniel, and N. Phillips. 1999. Low dietary protein during early pregnancy alters bovine placental development. Anim. Reprod. Sci. 55:13–21. doi: 10.1016/s0378-4320(98)00157-2 10099675

[CIT0033] Peter, A. T. 2013. Bovine placenta: a review on morphology, components, and defects from terminology and clinical perspectives. Theriogenology. 80:693–705. doi: 10.1016/j.theriogenology.2013.06.004 23849255

[CIT0034] Picking, E. M., L. A. Trubenbach, F. W. Bazer, J. E. Sawyer, T. A. Wickersham, and M. C. Satterfield. 2020. Technical note: Relationship between placentome location and gene expression in bovine pregnancy. J. Anim. Sci. 98:skaa176. doi: 10.1093/jas/skaa176 32452520 PMC7276672

[CIT0035] Prior, R. L., and D. B. Laster. 1979. Development of the bovine fetus. J. Anim. Sci. 48:1546–1553. doi: 10.2527/jas1979.4861546x 479048

[CIT0036] Rasby, R. J., R. P. Wettemann, R. D. Geisert, L. E. Rice, and C. R. Wallace. 1991. Nutrition, body condition and reproduction in beef cows: fetal and placental development and estrogen and progesterone in plasma. J. Anim. Sci. 68:4267–4276. doi: 10.2527/1990.68124267x 2286568

[CIT0038] Redifer, C. A., and A. M. Meyer. 2020. The relationships of late gestational uterine artery blood flow with calf and placental size. J. Anim. Sci. 98:48–49. doi: 10.1093/jas/skaa278.089 PMC11349610 38785319

[CIT0037] Redifer, C. A., N. B. Duncan, and A. M. Meyer. 2021. Factors affecting placental size in beef cattle: maternal and fetal influences. Theriogenology. 174:149–159. doi: 10.1016/j.theriogenology.2021.08.015 34454320

[CIT0040] Redifer, C. A., L. G. Wichman, A. R. Rathert-Williams, H. C. Freetly, and A. M. Meyer. 2023. Late gestational nutrient restriction in primiparous beef females: nutrient partitioning among the dam, fetus, and colostrum during gestation. J. Anim. Sci. 101:skad195. doi: 10.1093/jas/skad195 37314299 PMC10400126

[CIT0039] Redifer, C. A., L. G. Wichman, S. L. Davies, A. R. Rathert-Williams, H. C. Freetly, and A. M. Meyer. 2024a. Late gestational nutrient restriction in primiparous beef females: performance and metabolic status of lactating cows and pre-weaning calves. J. Anim. Sci. 102:skae015. doi: 10.1093/jas/skae015 38243834 PMC10894509

[CIT0041] Redifer, C. A., L. G. Wichman, A. R. Rathert-Williams, E. M. Shangraw, T. B. McFadden, and A. M. Meyer. 2024b. Nutrient restriction during late gestation reduces milk yield and mammary blood flow in lactating primiparous beef females. J. Anim. Sci. 102:skae016. doi: 10.1093/jas/skae016 38243877 PMC10898790

[CIT0042] Reid, D. S., D. D. Burnett, Z. E. Contreras-Correa, and C. O. Lemley. 2022. Differences in bovine placentome blood vessel density and transcriptomics in a mid to late-gestating maternal nutrient restriction model. Placenta 117:122–130. doi: 10.1016/j.placenta.2021.12.004 34883456

[CIT0045] Reynolds, L. P., and C. L. Ferrell. 1987. Transplacental clearance and blood flows of bovine gravid uterus at several stages of gestation. Am. J. Physiol. 253:R735–R739. doi: 10.1152/ajpregu.1987.253.5.R735 2825547

[CIT0046] Reynolds, L. P., C. L. Ferrell, D. A. Robertson, and S. P. Ford. 1986. Metabolism of the gravid uterus, foetus and utero-placenta at several stages of gestation in cows. J. Agric. Sci. 106:437–444. doi: 10.1017/s0021859600063309

[CIT0047] Reynolds, L. P., D. S. Millaway, J. D. Kirsch, J. E. Infeld, and D. A. Redmer. 1990. Growth and in-vitro metabolism of placental tissues of cows from day 100 to day 250 of gestation. J. Reprod. Fertil. 89:213–222. doi: 10.1530/jrf.0.0890213 1695680

[CIT0043] Reynolds, L. P., P. P. Borowicz, J. S. Caton, K. A. Vonnahme, J. S. Luther, D. S. Buchanan, S. A. Hafez, A. T. Grazul-Bilska, and D. A. Redmer. 2010a. Uteroplacental vascular development and placental function: an update. Int. J. Dev. Biol. 54:355–366. doi: 10.1387/ijdb.082799lr 19924632

[CIT0044] Reynolds, L. P., P. P. Borowicz, J. S. Caton, K. A. Vonnahme, J. S. Luther, C. J. Hammer, K. R. Maddock Carlin, A. T. Grazul-Bilska, and D. A. Redmer. 2010b. Developmental programming: the concept, large animal models, and the key role of uteroplacental vascular development. J. Anim. Sci. 88:E61–E72. doi: 10.2527/jas.2009-2359 20023136

[CIT0048] Robson, S. C., S. Hunter, R. J. Boys, and W. Dunlop. 1989. Serial study of factors influencing changes in cardiac output during human pregnancy. Am. J. Physiol. 256:H1060–H1065. doi: 10.1152/ajpheart.1989.256.4.H1060 2705548

[CIT0049] Rosenfeld, C. R. 1977. Distribution of cardiac output in ovine pregnancy. Am. J. Physiol. 232:H231–H235. doi: 10.1152/ajpheart.1977.232.3.H231 842676

[CIT0050] Short, R. E., and D. C. Adams. 1988. Nutritional and hormonal interrelationships in beef cattle reproduction. Can. J. Anim. Sci. 68:29–39. doi: 10.4141/cjas88-003

[CIT0051] Swanson, R., Z. Contreras-Correa, T. Dinh, H. King, D. Sidelinger, D. Burnett, and C. Lemley. 2022. Melatonin supplementation alters maternal and fetal amino acid concentrations and placental nutrient transporters in a nutrient restriction bovine model. Metabolites. 12:1208. doi: 10.3390/metabo12121208 36557248 PMC9782144

[CIT0052] Tanner, A. R., M. L. Bauer, K. C. Swanson, V. C. Kennedy, J. D. Kirsch, J. Gaspers, N. Negrin-Pereira, A. B. P. Fontoura, G. A. Perry, G. Stokka, et al. 2023. Influence of corn supplementation to beef cows during mid- to late-gestation: supplementation decreases placental microvascular surface density but does not alter uterine blood flow or neonatal performance. Livest. Sci. 268:105155. doi: 10.1016/j.livsci.2023.105155

[CIT0053] Thomas, J. W., and L. A. Moore. 1951. Variations in heart rate of dairy cows. J. Dairy Sci. 34:321–328. doi: 10.3168/jds.s0022-0302(51)91714-6

[CIT0054] Wallace, R. M., K. G. Pohler, M. F. Smith, and J. A. Green. 2015. Placental PAGs: gene origins, expression patterns, and use as markers of pregnancy. Reproduction. 149:R115–R126. doi: 10.1530/REP-14-0485 25661256

[CIT0055] Wichman, L. G., C. A. Redifer, and A. M. Meyer. 2023. Maternal nutrient restriction during late gestation reduces vigor and alters blood chemistry and hematology in neonatal beef calves. J. Anim. Sci. 101:skad342. doi: 10.1093/jas/skad342 37788576 PMC10648570

[CIT0056] Widdas, W. 1952. Inability of diffusion to account for placental glucose transfer in the sheep and consideration of the kinetics of a possible carrier transfer. J. Physiol. 118:23–39. doi: 10.1113/jphysiol.1952.sp004770 13000688 PMC1392425

[CIT0057] Wu, G., F. W. Bazer, J. M. Wallace, and T. E. Spencer. 2006. Board-invited review: intrauterine growth retardation: Implications for the animal sciences. J. Anim. Sci. 84:2316–2337. doi: 10.2527/jas.2006-156 16908634

[CIT0058] Young, M., and I. M. Fadyen. 1973. Placental transfer and fetal uptake of amino acids in the pregnant ewe. J. Perinat. Med. 1:174–182. doi: 10.1515/jpme.1973.1.3.174 4806571

